# Effects of glycerol and sorbitol on a novel biodegradable edible film based on *Malva sylvestris* flower gum

**DOI:** 10.1002/fsn3.3134

**Published:** 2022-11-17

**Authors:** Zeinab Jaderi, Fardieh Tabatabaee Yazdi, Seyed Ali Mortazavi, Arash Koocheki

**Affiliations:** ^1^ Department of Food Science, Faculty of Agriculture Ferdowsi University of Mashhad Mashhad Iran

**Keywords:** active packaging, *Malva sylvestris* flower gum, mechanical properties, packaging materials, young's modulus

## Abstract

There has been an increasing interest in the investigation of novel eco‐friendly packaging materials. An edible film based on *Malva sylvestris* flower gum was fabricated with 40%, 50%, and 60% glycerol and sorbitol using casting method. FTIR analysis was applied to identify the functional groups of films with different concentrations of plasticizers. The lightness of the samples did not affect upon increasing the sorbitol and glycerol; nevertheless, the samples tended to be green and yellow. SEM images indicated that glycerol‐based films enjoy heterogeneous and porous surfaces compared to the sorbitol‐based samples. Although Tensile Strength and Young's Modulus characteristics declined considerably (*p* < .05) upon plasticizer addition, elongation at break increased by more than 10% in glycerol‐based samples. A significant (*p* < .05) decrement was observed in the density of film strips via the addition of glycerol and sorbitol. Moisture content of films incorporated with both plasticizers saw a considerable improvement (*p* < .05) upon increasing the plasticizer concentration from 40% to 60% and is ascribed to the water‐holding capacity of plasticizers. Water contact angle and water solubility increased via plasticizer supplementation, which is attributed to the hydrophilic characteristic of glycerol and sorbitol, are in line with SEM analysis.

## Graphical abstract

In this research, a biodegradable edible film was produced using a Malva sylvestris flower gum. Afterwards, this research deals with screening the impacts of different concentrations of sorbitol and glycerol as plasticizers on the physicochemical properties of edible film.

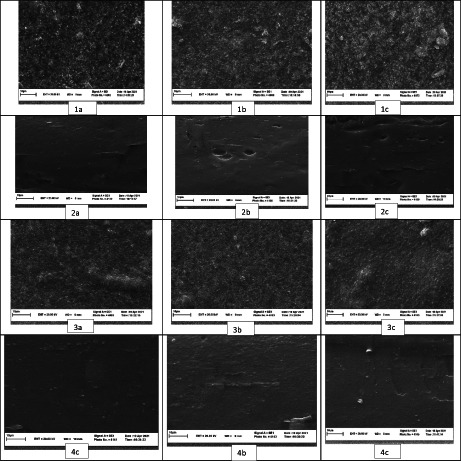

## 1 INTRODUCTION

The food safety concept comes at the top of the food industry and public health concerns. considering the improvements in technologies for producing food materials, still food pathogen contamination considered a threat to food sustainability and human health (Mukurumbira et al., 2022; Santos et al., 2017; Shirani et al., 2020). Packaging is a central vehicle for preserving food products against microbes, external contamination, light, and physical damage. Conventional packaging materials were based on fossil fuel resources like petroleum‐derived plastics. Nonetheless, the extensive use of synthetic plastics in packaging materials left many disadvantageous environmental effects due to its potential for persistency in the environment for several years and can develop microplastics or nanoplastics once it degrades that contaminate food products, soil, and water. Therefore, because of this method's many environmental side effects, there is an upward trend in a tendency toward bio‐based materials for food packaging which are entirely environmentally friendly (Pirnia et al., 2022; Sani et al., 2021). The innovative view of active packaging (AP) attracted researchers and manufacturers due to many promising attributes which lead to prolonged shelf‐life and warrant food quality and safety (Carpena et al., 2021). Not only are AP materials always provided from sustainable resources and will not affect the environment, but they also propose many hopeful properties like antioxidant, antimicrobial, and improved mechanical properties in many cases (Zhang et al., 2016). The application of biopolymers for this purpose is often valuable due to their potential in biodegradability and sustainability which make them eco‐friendly materials compared to the synthetic polymers (Iordanskii, 2020).

Several kinds of polysaccharides have differences in their molecular features that affect their physicochemical and biological attributes in packaging materials, including chitosan, chitin, cellulose, starch, and hydrocolloid gums, which have been applied for fabricating eco‐friendly films (Cazón et al., 2017; Sani et al., 2021). Polysaccharides are preferred among biopolymers due to their compatibility, higher stability, and the prospect of mixing with other biopolymers to reach the preferred mechanical properties. *Malva sylvestris* flower (MSF) constitutes a high‐molecular weight acidic polysaccharide (HMWAP) is 1.6 × 10^6^ D; uronic acids (22.8%) like glucuronic acid and galacturonic acid; and monosaccharides including 1,4‐Gal, 1‐GIcA, 1,3,4‐GalA, 1‐Gal, 1,2,4‐Rha, and 1,4‐Rha which were present in a molar ratio of 2.2:2.2:1.7:1.4:1.3:1.0 (Classen & Blaschek, 1998). It is also acknowledged as *Khabbazi* in Iran and be owned by the *Malvaceae* family widely found from the Mediterranean zone to central Asia. It was thought to show beneficial applications in treating inflammations of the uterus and kidney and dry cough traditionally (Al‐Snafi, 2013; Tabarsa et al., 2017; Yekta et al., 2020). Samavati & Manoochehrizade, 2013, found that crude polysaccharides of *M. sylvestris* leaves revealed considerably (*p* < .01) upper (89%) free radical‐scavenging activity in comparison to BHT (78%), indicating that it benefits from potential DPPH radical‐scavenging activity (Samavati & Manoochehrizade, 2013).

Modifying the mechanical characteristics of edible films that increase the elasticity, flexibility, and functionality of biopolymer films via diminishing the intermolecular forces and advancing the movement of polymer chains has been done using nonvolatile plasticizers, mostly polyols such as glycerol and sorbitol (Ahmadi et al., 2012; Dick et al., 2015). Several characteristics of glycerol, like low‐molecular weight, water solubility, and polarity, make it an appropriate plasticizer to be applied with a water‐soluble polymer (Dick et al., 2015).

It is documented that the different percentages of plasticizers influence the coating fabricating material. However, up to now, no detailed research has been performed to monitor the impact of different concentrations of glycerol and sorbitol on MSG films. This research aimed to screen the changes in appearance, mechanical properties, and barrier properties of MSG‐based films for providing a novel bio‐based active packaging.

## 2 MATERIALS AND METHODS

### 2.1 Materials

The *Malva sylvestris* applied was provided from a market at Khorasan‐Iran. Sorbitol and glycerol (analytical grade) were employed for film fabricating. Folin–Ciocalteu reagent, sodium carbonate, and 2, 2‐diphenyl‐1‐picrylhydrazyl (DPPH) were taken from Sigma Chemical Co (St. Louis, MO). Ethyl alcohol (≥99.7% CH_3_OH, 46.07 g/mol) was bought from tamad Kala Co, Iran. Additional materials were supplied from Merck Corporation, Germany.

### 2.2 Biopolymer film elaboration

Purification of polysaccharides from *Malva sylvestris* flower was completed after extraction. First, extraction of *M . sylvestris* powder was done considering the method of Yekta et al. (2020), using ethanol and then centrifugation (Yekta et al., 2020). Based on preliminary tests, 1.5 g of gum was used for film formation. To reach the homogenous dispersion, the gum was solubilized in distilled water by stirring at 65°C to reach the homogenous dispersion. Next, the solution was heated using a water bath at 80°C for 30 min and stirred at 120 rpm. Next, three levels of glycerol and sorbitol (40%, 50%, and 60%) as a plasticizer were added to the solution based on preliminary experiments. Then, the solution was homogenized by ultra‐turrax at 750 g for 5 min. Finally, the films were elaborated by casting procedure, and 25 g of the solution was poured into Petri dishes (with a diameter of 12 cm) and was placed in incubator at 30°C for 24 h. The experiments were performed in triplicate (Dick et al., 2015; Pirnia et al., 2022). Prior to determining mechanical properties, moisture content, and water vapor permeability, the herein films were stored in a desiccator at 25°C and 50% RH, 48 h in advance of the experiments.

### 2.3 Film characterization

#### 2.3.1 Fourier transform infrared spectroscopy (FTIR)

FTIR spectroscopy of biodegradable herein films was determined using an FTIR spectrometer (Thermo Nicolet; AVATAR‐370 FTIR). The spectra were assessed within a range of 400–4000 cm^−1^ using a resolution of 4 cm^−1^. An average of 32 scans has been mentioned for each sample. All measurements were performed at ambient temperature (Liang & Wang, 2018).

#### 2.3.2 Mechanical properties

Prior to the experiments, film samples were cut into the 2*7 cm^2^ strips and were left in a desiccator with Mg_2_NO_3_ to reach the RH 53%. Mechanical properties, including tensile strength (TS, MPa), elongation to break (EB, %), and Young's modulus (YM), and thickness of strips were assessed using an average of six replication by Instron Universal Testing Machine (Model A1 700; Gotech) based on methods of Dick et al. (2015).

(1) $$\text{Tensile strength}\left(\mathrm{MPa}\right)=\frac{F_{\max}}{W\times T}\;$$

(2) $$\text{Elongation to break}\;\left(\%\right)=\frac{L-L0}{A}\times 100$$

#### 2.3.3 Moisture content (MC) and water solubility (WS)

In order to measure the moisture content of herein films, an oven‐drying method at 105°C for 24 h was applied (Zareie et al., 2020), after which, the weight of the film strips remained constant.

(3) $$\mathrm{MC}=\left(\frac{w_{i}-w_{d}}{w_{i}}\right)\times 100\;$$

Water solubility (WS) was determined after heating the film strips using oven at 105°C for 24 h to reach stable dry weight using the method of Dick et al. (2015). WS% was calculated using the following equation:

(4) $$\text{Water}\;\text{solubility}\;\left(\%\right)=\frac{\text{initial}\;\mathrm{dry}\;\text{weight}-\text{final}\;\mathrm{dry}\;\text{weight}}{\text{initial}\;\mathrm{dry}\;\text{weight}}\times 100\;$$

#### 2.3.4 Water vapor permeability (WVP) and water contact angle (WCA)

The gravimetric method based on the ASTM method E96‐95 (1995) (Dick et al., 2015) with some modifications was applied in order to determine the WVP the film samples were enclosed in tubes containing anhydrous calcium chloride to keep the RH 0% and stages were followed based on the method of Pirnia et al. (2022).

(5) $$\text{WVTR}=\frac{S}{A}\;$$

(6) $$\mathrm{WVP}=\frac{\text{WVTR}\times X}{∆p}\;$$

*WVTR*: Water vapor transmission rate *S*: Slope of the regression model *A*: the permeation area (m^2^).

*X*: Film thickness (mm) *ΔP*: water vapor pressure difference across the film (KPa).

The value of WCA was determined using a Goniometer (Krüss Drop Shape Analyzer, Germany, Version 1.4.1.2) coupled with an image analysis software (Image J). WCA value used to measure surface hydrophobicity, performed through using the method of Pirnia et al. (2022).

#### 2.3.5 Color and light transmittance measurement

Color measurement of herein films was conducted using a colorimeter (Chroma meter CR‐410) and by analyzing the colorimetric parameters (a*, b*, and L*). L* stated for Lightness (0 for black to 100 for white), b* is an indication of yellow to blue, and a* used for range of red to green.

(7) $$∆E=\sqrt{{\left(∆L\right)}^{2}+{\left(∆a\right)}^{2}+{\left(∆b\right)}^{2}}$$

A light transmittance of herein films was detected using a UV–Visible spectrophotometer at 200–800 nm of wavelength (UV and Visible range) and using a below equation. Air was applied as a reference in the spectrophotometer. The higher value of transparency indicates the lower transparency of samples (Dick et al., 2015).

#### 2.3.6 Density and zeta potential

The strips of herein films (dimension of 3*3 cm^2^) were dried using a desiccator comprising P_2_O_5_(RH 0%) for a couple of weeks. The density of samples was recorded based on the following equation:

(8) $$\rho =\frac{\mathrm{dry}\;\text{weight of sample}}{\text{film thickeness}\times \text{surface area}}$$

The strips of herein films (dimension of 7*2 cm^2^) were dried using a desiccator comprising P_2_O_5_(RH 0%) for a couple of weeks (Yekta et al., 2020). The density of samples was recorded based on the method of Bi et al. (2021) and following equation (Bi et al., 2021):

#### 2.3.7 Scanning electronic microscopy (SEM)

The microstructure of herein film samples was considered using SEM images. In this regard, film samples have been cryofracture in liquid nitrogen and coated with thin gold for 5 min using a sputter coater. The surface and cross section of film strips were observed via a Scanning Electronic Microscopy apparatus at 20 kV and magnification of 4000 (Philips XL30, Netherlands) (Bi et al., 2021; Pirnia et al., 2022).

#### 2.3.8 Statistical analysis

All measurements of this study were applied as mean values and standard deviation (SD). One‐way analysis of variance (ANOVA) and the Duncan test with *α* = .05 were completed by SPSS software.

## 3 RESULTS AND DISCUSSION

### 3.1 Characterization of edible films (FTIR, density, and zeta potential)

**FIGURE 1:**
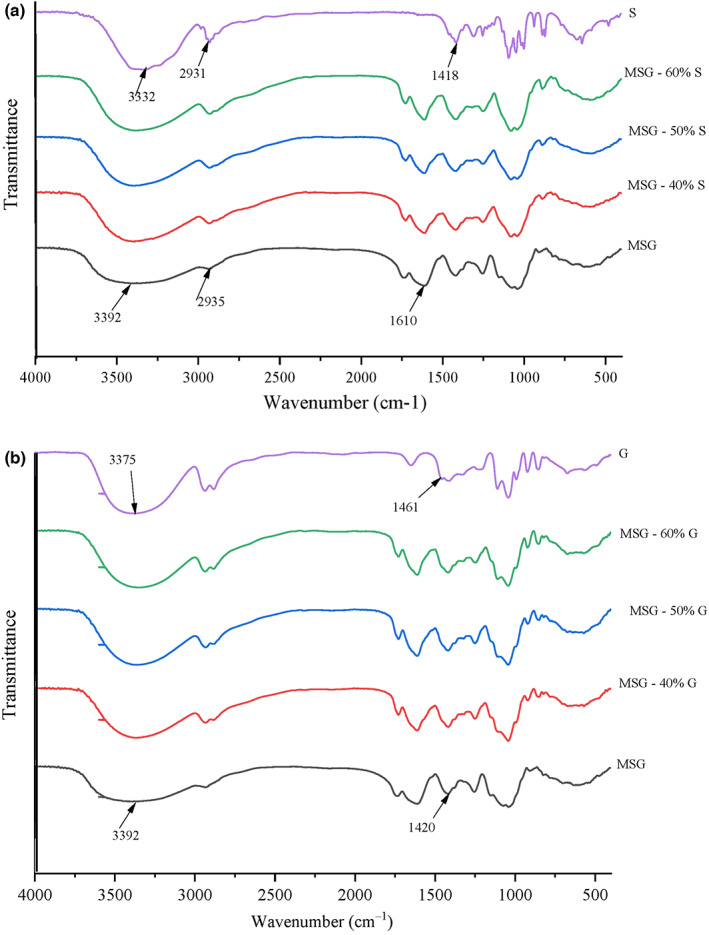
(a):FTIR spectra of MSG films incorporated with sorbitol. (b): FTIR spectra of MSG films incorporated with glycerol.

FTIR spectra of G, S, MSG‐based edible films containing different concentrations of sorbitol and glycerol (MSGS and MSGG) are provided in Figure 1, A, B, respectively. According to Figure 1, for MSG films, the broad absorption band at 3392 cm^−1^ is attributed to O‐H stretching vibration, which is formed due to the hydrogen bonding of glucopyranose O‐H groups. A peak of 2800–3000 cm^−1^ was ascribed to C‐H symmetric and asymmetric vibrations. A peak at 1610 cm^−1^ could be formed due to the C=O stretching vibration in MS gum and arise from uronic acid residues. The several peaks in the range of 1000–1500 cm^−1^ mostly appeared due to the bending vibration of groups including COC, COH, HCH, HCO, and CCH. A higher number of absorption bands in the range of 800–1200 cm^−1^ represents the presence of monosaccharides, including rhamnose, galactose, glucose, arabinose, and mannose, which is most significant in the analysis of polysaccharides.

Furthermore, a band around 1100 cm^−1^ might represent C‐O‐C glycosidic ether. The band shifts in films incorporated with sorbitol and glycerol are ascribed to the intermolecular hydrogen bands contributing to sorbitol and glycerol and film matrices. These new intermolecular bands will affect the film structure, flexibility, compactness, and homogeneity. The bands' intensity and width in 3500–2930 cm^−1^ were improved in films loaded with sorbitol and glycerol, illustrating new hydrogen bands formation among sorbitol and glycerol and MS gum (Yekta et al., 2020). Peaks in the 800–1500 cm^−1^ correspond to the stretching of C‐O‐C that arose from glycosidic bodies and O‐H stretching vibration of the pyranose functional group in gum. The FTIR spectra show that all the films loaded with glycerol and sorbitol have similar patterns. Results indicate that MSG has good compatibility with glycerol and sorbitol. The spectra of glycerol and sorbitol are primarily similar in the case of absorption band areas. In line with our study, other researchers also found that the addition of sorbitol and glycerol will increase the intermolecular hydrogen bonds (Cao et al., 2018; Dick et al., 2015).

A gradual decrement was observed in the density of film strips via the addition of glycerol and sorbitol, which was deduced significantly (*p* < .05). It can be ascribed to the lower density of higher concentrations of sorbitol and glycerol and the interactions they formed with biopolymer film matrices. The decrease in density might be attributed to the increased thickness (and volume) associated with the increment of the plasticizer portion. Razavi et al. (2015) also found a density decrement via raising the portion of plasticizers (Razavi et al., 2015).

Zeta potential is an identification of interfacial charge of emulsions. The zeta potential of 1.5% MSG in deionized water was identified and reported as −52.62 mv, which is due to the ionic character of MSG. Particles with an average of zeta potential higher than 30 mv are generally considered stable, which is ascribed to the strong repulsive forces to ensure the satisfactory dispersion of nanoemulsions (Jiang et al., 2015).

### 3.2 SEM observation

**FIGURE 2:**
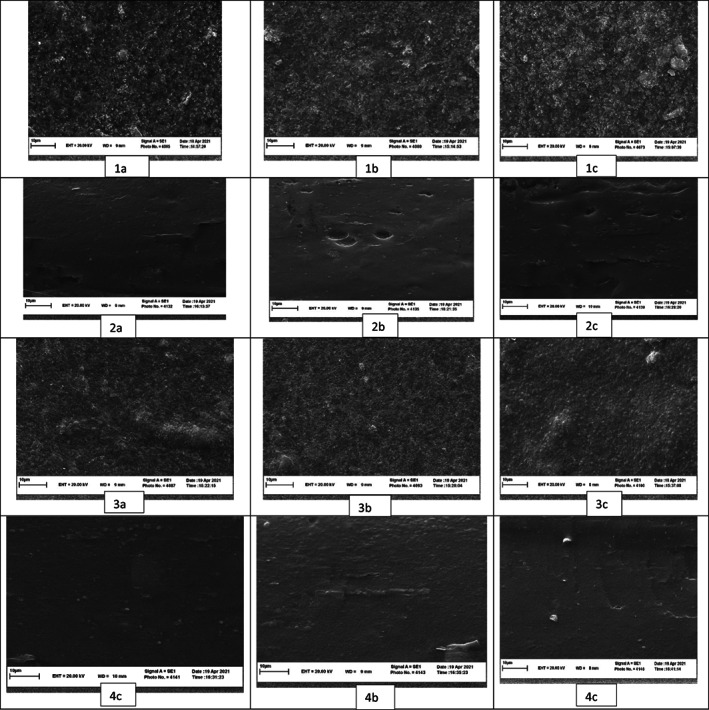
SEM images of glycerol‐loaded films surface (1A:40%, 1B:50%, 1C:60%) and cross images (2A:40%, 2B:50%, 2C:60%). Sorbitol‐loaded films surface (3A:40%, 3B:50%, 3C:60%), and cross images (4A:40%, 4B:50%, 4C:60%)

Images of scanning electron microscopic observation are shown in Figure 2, illustrating the microstructure of the cross section and surface of herein film with 40%, 50%, and 60% glycerol and sorbitol. Microscopic views indicated a relatively heterogeneous and rough surface. Nonetheless, the changes via increasing the concentration of glycerol and sorbitol are not remarkable. The cross section images show that film loaded with sorbitol enjoys smooth, homogenous, and coherent structure and texture compared to the glycerol‐loaded biopolymer films (Liang & Wang, 2018; Pirnia et al., 2022). Improving the concentration of glycerol leads to an increase in the presence and number of porous which is evident in cross section images.

### 3.3 Color and light transmission analysis

**FIGURE 3:**
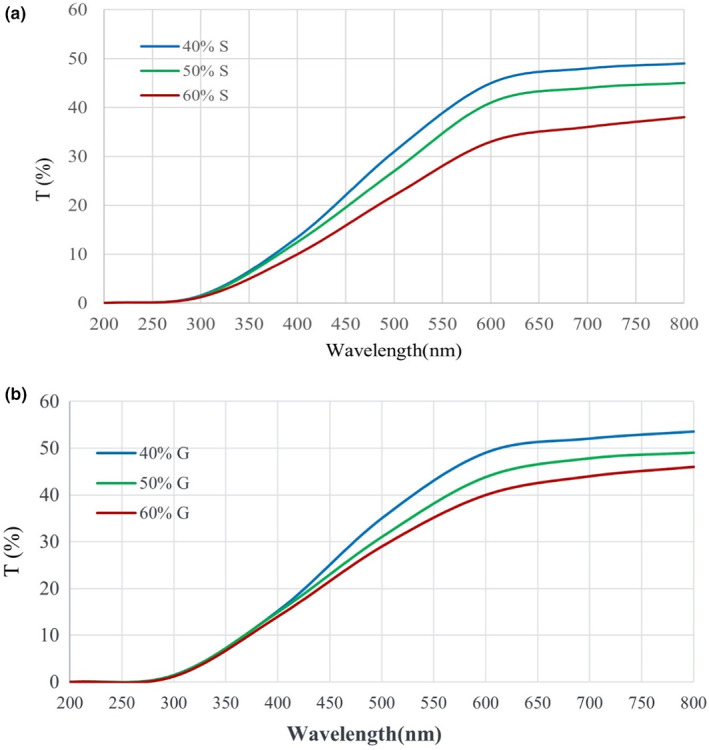
Light transmittance curves of films loaded with sorbitol (a) and glycerol (b)

The light transmission spectra of herein films incorporated with different concentrations of sorbitol and glycerol are provided in Figure 3 a and b, which indicate a significant difference in light transmission in different concentrations. There is no significant difference via increasing glycerol concentration at 200–400 nm. The transmittance value for films with 40%, 50%, and 60% of glycerol was 52.1%, 47.8%, and 44.3%, indicating a significant decrease via the addition of glycerol. In the case of the sorbitol curve, it is noticeable that the transmission was similar for each concentration until 300 nm. After that, there is a significant change in light transmission of different concentrations. Like the glycerol trend, the MSG films incorporated with 60% sorbitol exhibited the lowest transmission among 40%, 50%, and 60% sorbitol. The results indicated that MSG films became gradually opaque via improving the sorbitol and glycerol content. Also, it indicates that it has the potential ability to show acceptable UV barrier characteristics against oxidation, off‐flavors of foodstuffs, decolorizations, and nutrient element loss. In line with our findings, Zhang et al. (2016) reported similar results in gum *ghatti* films and claimed that this phenomenon could be ascribed to the dispersion of sorbitol and glycerol drops throughout the films, which cause breakdown in the film structure. The results show that the films could offer promising attributes in case of shielding against the light (Cao et al., 2018; Zhang et al., 2016).

Based on color changes results which are presented in Table.1. It can be deduced that the film's lightness was increased upon the increase of glycerol and sorbitol, but the changes were not significant. It is associated with the transparent appearance of films, as well. Also, the significant decrease of a* in films loaded with glycerol shows that the greenness of samples tends to increase via improving the glycerol from 40% to 60%. However, the changes in a* value were not deduced significantly for sorbitol‐loaded samples. The changes in b* indicated that yellowness in samples with sorbitol decreased significantly via sorbitol addition. The film's appearance generally has semi‐light green and yellow severity, which is associated with the gum composition and its reactions with high content of sorbitol and glycerol. Dick et al. (2015) reported that chia seed mucilage films with a high glycerol content were slightly reddish and yellowish, but they remained transparent (Dick et al., 2015).

**TABLE 1:** Density, color parameters, and water vapor permeability (WVP) of MSG films

Plasticizer (%, w/w MSG)	Density (g/cm^3^)	Color parameters				*WVP* (×10^−11^ g/m s Pa)
		*L* ^*^	a*	b*	*ΔE*	
Glycerol						
40	1.40 ± 0.01 ^a^	72.79 ± 0.15 ^a^	−1.50 ± 0.14 ^a^	29.59 ± 0.14 ^a^	35.27 ± 0.19 ^a^	1.81 ± 0.49 ^bc^
50	1.38 ± 0.01 ^a^	73.76 ± O.11 ^a^	−2.64 ± 0.01 ^b^	27.95 ± 0.06 ^cba^	33.39 ± 0.11 ^ab^	3.15 ± 0.05 ^a^
60	1.32 ± 0.05 ^b^	75.04 ± 0.62 ^a^	−2.71 ± 0.13 ^b^	26.70 ± 0. 41 ^a^	31.65 ± 0.68 ^b^	3.61 ± 0.15 ^a^
Sorbitol						
40	1.38 ± 0.05 ^a^	73.81 ± 0.85 ^a^	−2.53 ± 0.15 ^c^	29.88 ± 0.76 ^a^	35.01 ± 1.10 ^a^	0.82 ± 0.11 ^d^
50	1.31 ± 0.02 ^b^	74.05 ± 2.76 ^a^	−3.22 ± 0.02 ^c^	29.10 ± 1.13 ^cb^	34.33 ± 0.45 ^a^	1.32 ± 0.11 ^cd^
60	1.26 ± 0.01 ^c^	76.09 ± 0.06 ^a^	−3.32 ± 0.01 ^c^	27. 25 ± 0. 28 ^bc^	31.53 ± 0.28 ^b^	2.71 ± 0.39 ^ab^

*Note*: Means within each column with different superscripts are significantly different (*p* < .05).

Abbreviations: MSG is *Malva sylvestris* gum; ΔE is total color difference; WVP is water vapor permeability (×10^−‐11^ g/m s Pa).

### 3.4 Mechanical properties and thickness

The thickness of synthesized samples is considered one of the vital characteristics due to its influence on other factors, including barrier properties against water, optical, and mechanical characteristics. As is shown in Table.2, the thickness of samples loaded with glycerol increased, but the changes were not significant. Meanwhile, the thickness of sorbitol‐loaded films increased significantly upon the addition of sorbitol from 40% to 60%.

**TABLE 2:** Thickness, moisture content, solubility, and water contact angle of MSG films

Plasticizer (%)	Thickness (mm)	MC (%, d.b.)	WS (%)	Water contact angle (°)
Glycerol				
40	0.071 ± 0.00 ^ab^	18.53 ± 0.96 ^c^	69.52 ± 0.85 ^c^	86.05 ± 0.75 ^a^
50	0.080 ± 0.001 ^a^	29.43 ± 0.83 ^b^	77.20 ± 0.47 ^b^	75.7 ± 0.20 ^b^
60	0.087 ± 0.001 ^a^	38.97 ± 1.17 ^a^	83.74 ± 1.73 ^a^	73.7 ± 1.80 ^bc^
Sorbitol				
40	0.078 ± 0.003 ^b^	13.06 ± 0.56 ^e^	64.79 ± 0.83 ^d^	76.25 ± 0.75 ^b^
50	0.086 ± 0.002 ^a^	15.21 ± 0.31 ^de^	71.09 ± 0.71 ^c^	70.95 ± 0.55 ^c^
60	0.098 ± 0.001 ^b^	16.42 ± 0.52 ^cd^	77.78 ± 0.57 ^b^	62.81 ± 0.58 ^d^

*Note*: Means within each column with different superscripts are significantly different (*p* < .05).

Abbreviations: MC, moisture content in dry basis (%); MSG, *Malva sylvestris* gum; WS is water solubility (%).

Incorporation of glycerol and sorbitol on the mechanical characteristics of MSG films synthesized at 25°C, and 52% RH, considering the YM, EB, and TS are revealed in Figure 3. The improvement in the concentration of sorbitol and glycerol caused a remarkable change (*p* < .05) in mechanical characteristics, which are studied in this research. As shown, although TS and YM saw a significant decrease (*p* < .05) upon the addition of sorbitol and glycerol, the EB improved by increasing the content of two plasticizers. The application of plasticizers in film synthesizing is associated with modifying the mechanical properties by increasing polymer chains' mobility and reducing the intermolecular forces. Although the YM and TS of samples loaded with sorbitol were generally higher than that of glycerol, the EB % was higher in cases synthesized with glycerol. The percent of EB increased as a function of glycerol and sorbitol in herein films while TS and YM decreased significantly (*p* < .05) via increasing the sorbitol and glycerol. The physical cross‐linking, which is ascribed to the solid intermolecular linking between gum and plasticizers, leaves a decreased effect on the free volume and the molecular migration of MSG polymer. The decrement of TS might be associated with the combination of sorbitol and glycerol with MSG matrices, which deteriorated the cross‐linking of MSG and improved the free volume. The increasing impact of plasticizers on EB could be ascribed to the efficient transfer of participated stress throughout the polymer layers caused by intense chemical interactions between functional groups of gum (hydroxyl and carboxyl groups) and plasticizers (Yekta et al., 2020). In agreement with our findings, previous studies reported similar results via the addition of sorbitol and glycerol to biopolymer film matrices (Cao et al., 2018; Dick et al., 2015).

**FIGURE 4:**
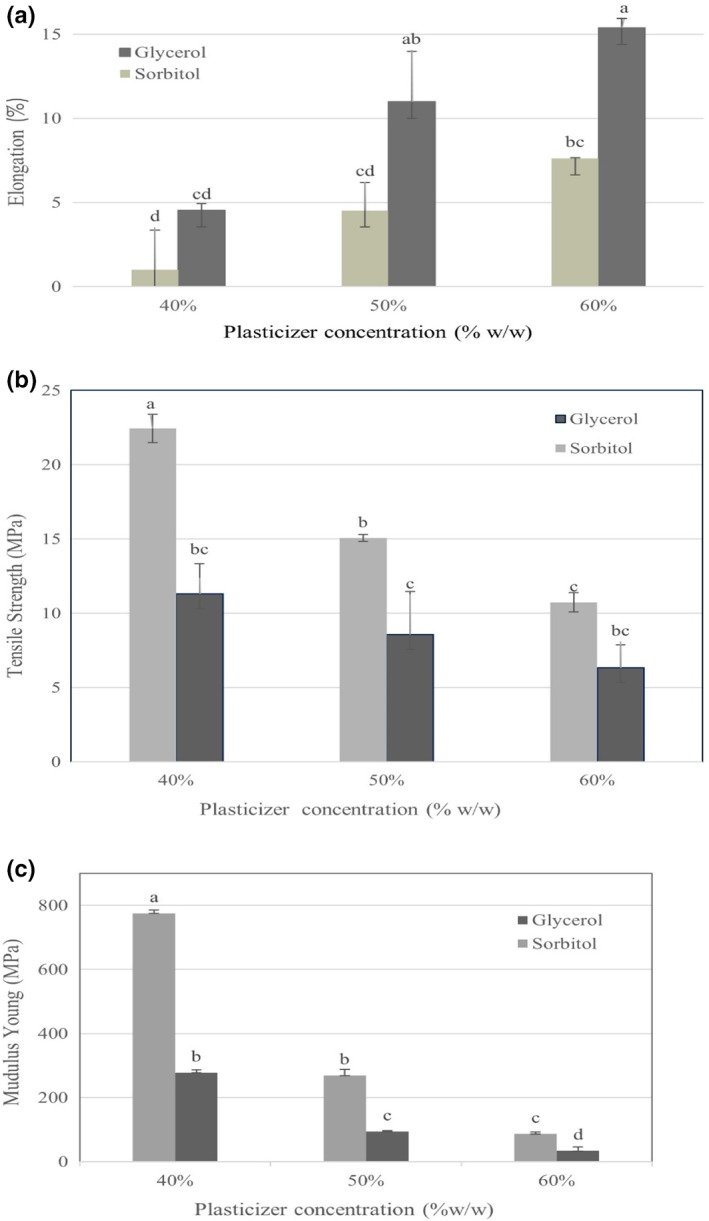
Elongation at break (a), tensile Strength (b), and Young's Modulus (c) of films via addition of sorbitol and glycerol

Concerning Young's Modulus (YM) or elastic modulus, which is associated with the stiffness of materials, the optimum stiffness is indicated by the higher value of YM. As it is evident in Figure 4, the impact of plasticizer content on the YM (MPa) of herein films is like their correspondent TS (MPa). The YM of films experienced a significant reduction via increasing plasticizer incorporation. It can be explained by the modifying role of plasticizers in film matrices, promoting the extension of hydrogen bonds between the substitutes, and weakening the solid intermolecular connections. Therefore, the YM indices and rigidity of synthesized films decreased significantly (*p* < .05)(Ibrahim et al., 2019). Similar results were reported by Dick et al. (2015) who found that the concentration of glycerol in films caused significant differences (*p* < .05) in TS, EB, and YM values. Improving the concentration of glycerol in the CM films from 25% w/w to 75% w/w decreased TS and YM and increased EB. Cao et al. (2018) fabricated the film based on cassia gum with glycerol and reported that films had EB than sorbitol loaded films. Additionally, TS increased with increasing glycerol (except for 50%) and sorbitol concentrations (Cao et al., 2018).

### 3.5 WVP, MC, WS, and WCA measurement

Moisture content (MC) of films incorporated with both plasticizers saw a considerable improvement (*p* < .05) upon increasing the plasticizer concentration from 40% to 60%, which causes increasing the water retention in film, and it is ascribed to the water‐holding capacity of plasticizers. The difference in water retention of glycerol and sorbitol is associated with intermolecular linkages and molecular structure of plasticizers that can improve the chance of plasticizers interacting with water molecules (Ibrahim et al., 2019). As shown in Table.2, the MC value of 60% glycerol‐based film was the highest, attributed to the above reasons.

Water solubility (WS) is a crucial factor in packaging materials in cases when water insolubility, water resistance, and product integrity are essential. Based on the results provided in Table.2, the water WS increased remarkably (*p* < .05) via plasticizers addition. Considering the hydrophilic nature of plasticizers, the increase in the concentration of plasticizers reduces interactions of polymer molecules. It improves the free spaces in polymer matrices, affecting the water penetration into the film matrices and causing a significant increase in water solubility of samples loaded with a higher concentration of plasticizers (Edhirej et al., 2017; Sanyang et al., 2016). Generally, the higher MC and WS of glycerol‐based films are ascribed to their heterogeneous structure, making it potent to retain more water in the film structure.

Based on the data presented in Table.1, WVP increased significantly (*p* < .05) via addition of plasticizers from 40% to 60%. The WVP of films incorporated with 60% glycerol was higher than that of sorbitol. Based on the research of Bi et al. (2021) the denser structure might reduce the free volume in film matrices and thereby hamper the path of water vapor (Bi et al., 2021). Therefore, the lower WVP in films loaded with 40% sorbitol and glycerol is attributed to the higher density observed in synthesized films with 40% glycerol and sorbitol.

Additionally, the WVP of films containing sorbitol is lower than that of glycerol. It is attributed to the compact and dense structure of sorbitol‐based films compared to the glycerol ones observed in SEM images. Meanwhile, the glycerol‐loaded films enjoy a more heterogeneous and porous structure which can increase their potential for retention of water molecules.

One of the critical characteristics of packaging materials is their attributes related to hydrophobicity, hydrophilicity, and wettability of films for further application in industry. Generally, the WCA of <90°C is an attribute of hydrophilic materials (Cao et al., 2018; Pirnia et al., 2022). WCA is an indicator of the determination of surface tension characterization, which is used to measure the penetration and evaporation of water in film matrices. Regarding the data presented in Table.2, there is a significant (*p* < .05) decrease in WCA from 86.05°C to 73.7°C for glycerol‐based films and from 76.25°C to 62.81°C for sorbitol‐loaded films upon the increasing plasticizer's concentration from 40% to 60%. It is well documented that WCA decreases via increasing the surface hydrophilicity; thereby, WCA decrement as a function of plasticizers addition is attributed to the hydrophile nature of plasticizers. It might be ascribed to the hydrogen bonds formed between sorbitol, glycerol, and polymer matrices. Considering the information provided for previous water‐related and morphology information, observed WCA data are entirely in line with MS, WC, WVP, and SEM images. Cao et al. (2018) reported similar results via observing the effect of sorbitol and glycerol on the contact angle of Cassia gum‐based films (Cao et al., 2018).

## 4 CONCLUSION

The *Malva sylvestris* gum is an interesting ingredient for the fabrication of new coating solutions, and this research demonstrated that MSG edible films plasticized with glycerol and sorbitol can be prepared successfully. The impacts of three different concentrations of glycerol and sorbitol were on properties of herein films were screened. Based on the results, although the YM and TS of samples loaded with sorbitol were higher than that of glycerol, the EB % was higher in samples synthesized with glycerol. It was deduced that although the addition of plasticizers did not affect the lightness of samples, the greenness and yellowness of films changed significantly. The MC, WS, WCA, and WVP of films were increased significantly as a function of plasticizers addition due to the hydrophilic nature of sorbitol and glycerol and are attributed to the formation of hydroxyl groups and are in line with SEM images. Considering all, films synthesized with glycerol revealed desirable mechanical properties for use in food packaging. These films can be integrated with antimicrobial components (e.g., essential oils) for antimicrobial edible packaging purposes.

## ETHICAL APPROVAL

This study was approved by the Institutional Review Board of the Ferdowsi University of Mashhad.

## AUTHOR CONTRIBUTIONS

**Zeinab Jaderi** involved in conceptualization, methodology, resources, investigation, formal analysis, data curation, writing—original draft, writing—review & editing. **Farideh Tabatabaee Yazdi** involved in funding acquisition, project administration, supervision, writing—review & editing. **Seyed Ali Mortazavi** involved in supervision and funding acquisition. **Arash Kocheki** involved in formal analysis, data curation, and project administration.

## CONFLICT OF INTEREST

The authors declare no conflicts of interest.

## Data Availability

Data will be provided under the request.
